# Prevalence and Serotype Distribution of Foot and Mouth Disease (FMD) Virus in Asian Countries: A Systematic Review and Meta‐Analysis

**DOI:** 10.1155/vmi/5492291

**Published:** 2026-02-09

**Authors:** Md Jisan Ahmed, Md Imran Hossain, Md Arifur Rahman, Md Ismile Hossain Bhuiyan, Prajwal Bhandari, Kazi Estieque Alam, Ritu Chalise, Israt Jahan Kaderi, Md Afiqul Islam Rahi, Tashmiah Tarin, Md Jahangir Alam, Ridwan Olamilekan Adesola, Delower Hossain

**Affiliations:** ^1^ Association of Coding, Technology, and Genomics (ACTG), Sher-e-Bangla Agricultural University (SAU), Dhaka, 1207, Bangladesh, sau.edu.bd; ^2^ Department of Pathology, Faculty of Animal Science and Veterinary Medicine, Sher-e-Bangla Agricultural University (SAU), Dhaka, 1207, Bangladesh, sau.edu.bd; ^3^ Department of Surgery and Theriogenology, Faculty of Animal Science and Veterinary Medicine, Sher-e-Bangla Agricultural University (SAU), Dhaka, 1207, Bangladesh, sau.edu.bd; ^4^ Department of Animal Production and Management, Faculty of Animal Science and Veterinary Medicine, Sher-e-Bangla Agricultural University (SAU), Dhaka, 1207, Bangladesh, sau.edu.bd; ^5^ Department of Microbiology and Parasitology, Faculty of Animal Science and Veterinary Medicine, Sher-e-Bangla Agricultural University (SAU), Dhaka, 1207, Bangladesh, sau.edu.bd; ^6^ Department of Anatomy, Histology, and Physiology, Faculty of Animal Science and Veterinary Medicine, Sher-e-Bangla Agricultural University (SAU), Dhaka, 1207, Bangladesh, sau.edu.bd; ^7^ Department of Veterinary Medicine and Surgery, University of Missouri, Columbia, Missouri, USA, missouri.edu; ^8^ Department of Medicine and Public Health, Faculty of Animal Science and Veterinary Medicine, Sher-e-Bangla Agricultural University (SAU), Dhaka, 1207, Bangladesh, sau.edu.bd; ^9^ Department of Veterinary Medicine and Animal Sciences, Università Degli Studi di Milano, Lodi, 26900, Italy, unimi.it

**Keywords:** Asia, epidemiology, FMD, meta-analysis, prevalence, serotypes

## Abstract

**Background:**

Foot and mouth disease (FMD) is a highly contagious transboundary List A infectious disease of domesticated and wild cloven‐hoofed animals, causing considerable economic impact through production losses and trade bans on livestock.

**Objective:**

This study aimed to determine the pooled prevalence of FMD and the distribution of FMD virus serotypes identified in Asian countries from 2008 to 2025.

**Methods:**

A systematic search for studies on the prevalence of FMD in domesticated and wild animals was performed via PubMed, SpringerLink, Google Scholar, and ScienceDirect, published between January 1, 2008, and March 1, 2025. Studies were selected according to the established inclusion and exclusion criteria. The pooled prevalence was estimated using a random‐effect meta‐analysis model in Stata v18.0 because significant heterogeneity was observed across studies.

**Results:**

The overall pooled prevalence of FMD in Asian countries was 42.43% (95% CI: 35.55%–49.46%), with significant variation across species, countries, diagnostic techniques, years, and disease occurrence. Buffalo (74.30%) was the most affected species, followed by sheep (54.95%), cattle (51.6%), pigs (43.83%), and goats (15.62%). The highest pooled estimates were observed for outbreaks (51.48%), RT‐PCR (54.13%), Bangladesh (72.86%), and vesicles as sample types (96.77%). Among the seven (7) serotypes, serotype O is prevalent in Asia, followed by A and Asia‐1. Although rare in the region, SAT‐2 has been detected only in Iraq among Asian countries.

**Conclusion:**

FMD is endemic in most Asian countries. FMD, a List A disease, severely impacts the international trade of live animals and animal products due to its transboundary nature. Effective prevention, control, and eradication strategies, including strengthened surveillance, timely reporting, and the development of multivalent vaccines to achieve global FMD eradication, are essential.

## 1. Introduction

Foot and mouth disease (FMD) is a transboundary and trade‐sensitive disease that affects livestock production [1–3]. It represents a significant economic challenge, particularly in endemic regions. Globally, the disease causes economic losses ranging from US$6.5 to $21 billion annually in endemic areas, with FMD‐free countries and zones also incurring costs exceeding US$1.5 billion per year [2, 4]. Owing to its severe impact, the World Organization for Animal Health (WOAH) declared FMD a List A disease and ranks first among animal infectious diseases [5].

Foot and mouth disease virus (FMDV) is the causative agent of FMD and is a single‐stranded positive‐sense ribonucleic acid (RNA) virus in the family *Picornaviridae* and genus *Aphthovirus*. The virus appears as seven major serotypes, viz. O, A, C, Asia‐1, SAT‐1 (Southern African Territory), SAT‐2, and SAT‐3, which offer no cross‐immunity against each other [3, 6]. With each of these serotypes, serologically, there are distinct subtypes with varying degrees of virulence due to their infinite mutation rates [7]. It results in the appearance of new, antigenically different subtypes that are distributed around the world [8]. Among the seven (7) serotypes, four (4) serotypes of FMD viruses, O, A, Asia‐1, and SAT‐2, have been reported in Asian countries, where serotype O is the most prevalent [9, 10]. In Bangladesh, surveillance of 71 outbreaks between 2012 and 2021 reported an overall FMD prevalence of 54.7%, with serotype O predominating (85% of outbreaks), followed by serotype A (11%) and Asia 1 (4%) [11]. Moreover, an epidemiological investigation in Pakistan documented an overall FMDV prevalence of 33.2% among livestock [12]. Similarly, studies from India have demonstrated heterogeneous serotype distribution, with serotypes O, A, and Asia 1 accounting for approximately 43%, 11.6%, and 31.5%, respectively, highlighting substantial regional variation in the epidemiology of FMD across the country [13]. The serotypes of FMDV immunologically differ from each other; hence, vaccines prepared from one serotype do not protect against other serotypes [14]. FMD remains endemic across the majority of Asian countries, posing a significant economic threat to the region. However, Indonesia and the Philippines are exceptions, as they are recognized as FMD‐free nations within Asia [15].

FMD is a contagious disease that affects cloven‐footed domestic animals, including cattle, sheep, goats, pigs, and camels, and buffaloes and wild animals, such as gayal and oryx, and poses a severe threat to the livestock industry [3]. It is characterized by fever, followed by vesicles and erosions on the tongue, gums, lips, interdigital space, mammary glands, and other glabrous skin parts, leading to lameness and other signs, including excessive salivation and anorexia [16, 17]. Susceptible animals are infected directly via direct contact with respiratory aerosols and droplets from infected animals or indirectly via the environment or mechanically by persons, vehicles, wild animals, or birds [18]. The vesicles often join to form enormous, inflated blisters that burst, leaving raw, painful ulcers behind. Although FMD has a low mortality rate in adult animals, it has debilitating symptoms such as weight loss, decreased milk production (up to 33%), reproductive failure, and loss of draught power, which ultimately lower productivity and result in economic losses. Among susceptible populations, the infection rate reaches 100%. In young suckling calves, the fatality rate is also 100%, with deaths attributed primarily to myocarditis (known as tiger heart disease), which can account for up to 50% of mortality cases [19].

FMD prevention relies heavily on early detection through accurate diagnosis and effective surveillance [20]. Control strategies implemented to curb the spread of the disease include aphthization, movement restrictions on animals and animal products, quarantine, vaccination, the enforcement of biosafety and biosecurity measures, culling, stamping, and public awareness campaigns focused on prevention and control practices [3]. The Progressive Control Pathway for Foot and Mouth Disease (PCP‐FMD), developed by the Food and Agriculture Organization (FAO) and European Commission for the Control of Foot‐and‐Mouth Disease (EuFMD) and endorsed by WOAH, is a risk‐based, stepwise tool for FMD control in endemic countries. It categorizes countries into stages (0–3) on the basis of FMD risk and control progress, serving as a core component of the Global FMD Control Strategy alongside the Performance of Veterinary Services (PVS) framework of WOAH [21].

To the best of our knowledge, there is no review or meta‐analysis of the prevalence of FMD in Asia, despite the availability of several narrative and country‐specific reviews on FMD in Asia. Moreover, a substantial proportion of the existing literature predates recent changes in animal movement dynamics, vaccination programs, and surveillance capacity across the region. In the context of an evolving epidemiological landscape, intensified transboundary livestock trade, and renewed regional disease control initiatives, an updated quantitative synthesis is critically needed to generate robust regional estimates, identify persistent knowledge gaps, and support evidence‐based policy formulation and targeted control strategies. Furthermore, meta‐analysis is considered an innovative technique for estimating livestock disease prevalence and is not widely used in veterinary sciences [22]. Therefore, this systematic review and meta‐analysis were conducted to determine the prevalence of FMD in Asian countries and the distribution of FMDV serotypes.

## 2. Materials and Methods

### 2.1. Guidelines and Protocol

The study adhered to the PRISMA (Preferred Reporting Items for Systematic Reviews and Meta‐Analyses) guidelines for conducting a systematic review and meta‐analysis. To ensure the inclusion of all relevant information and uphold methodological standards, the PRISMA 2009 checklist was followed (Supporting Information S1). The methodological framework of this study was adapted from our previously published work [23], with minor modifications to suit the objectives of the present investigation.

### 2.2. Literature Search Strategy and Selection

To identify research on the prevalence of FMD in Asia, a comprehensive systematic literature search was conducted in multiple electronic databases, including PubMed, Google Scholar, Springer Link, and Science Direct, covering the period from January 2008 to February 2025. The search was conducted on January 8, 2025. The database search was conducted with filters based on the inclusion and exclusion criteria to ensure relevance. The following search strings were used to obtain the appropriate literature: “Prevalence” OR “Seroprevalence” OR “Incidence” OR “Frequency” OR “Occurrence” OR “Characterization” OR “Epidemiology” AND “FMD” OR “Foot and Mouth Disease” (Table S4).

### 2.3. Inclusion and Exclusion Criteria

For this systematic review and meta‐analysis, specific inclusion and exclusion criteria were established to ensure the selection of relevant, high‐quality studies on the prevalence of FMD across various species in Asian countries. The included studies were peer‐reviewed, English‐language articles reporting primary research conducted between January 1, 2008, and March 1, 2025; included retrospective, prospective, and cross‐sectional studies that presented the prevalence of FMD with endemic, epidemic, or outbreak occurrence; were conducted using valid screening methods, such as PCR, RT‒PCR, and ELISA; and provided a total sample size and the number of positive cases. Similarly, articles that did not meet the inclusion criteria were excluded from the study.

### 2.4. Data Extraction

Eight authors (Md Imran Hossain, Md Arifur Rahman, Md Ismile Hossain Bhuiyan, Prajwal Bhandari, Ritu Chalise, Israt Jahan Kaderi, Md Afiqul Islam Rahi, and Tashmiah Tarin) independently worked on each stage of the review process, including screening titles and abstracts, reviewing full texts, extracting data, and performing quality assessment. They tabulated the relevant data systematically from eligible studies in Microsoft Excel. The following data were extracted from each included study: country, author, publication year, species, types of samples tested, sample size, positive samples, detection method, disease occurrence, and serotypes of FMDV (Table 1). Discrepancies in study selection or data extraction were resolved through discussion between the authors, and when necessary, a third senior author (Delower Hossain) was consulted to reach a final decision.

**TABLE 1:** Summary of metadata of FMD from 2008 to 2025 published studies in Asia.

Country	Author	Species	Types of samples	Sample size	Positive sample	Detection method	Disease occurrence
Afghanistan	[24]	Cattle, buffalo, and sheep	Blood	7558	4089	ELISA	Outbreak
Afghanistan	[25]	Cattle	Blood	376	158	ELISA	Endemic
Afghanistan	[26]	Cattle and buffalo	Swab	180	22	RT‐PCR	Endemic
Bangladesh	[27]	Cattle	Epithelium	134	98	RT‐PCR	Outbreak
Bangladesh	[9]	Cattle	Epithelium	56	38	RT‐PCR	Outbreak
Bangladesh	[10]	Cattle and pig	Epithelium	304	206	RT‐PCR	Outbreak
Bangladesh	[11]	Cattle, buffalo, and pig	Epithelium	481	230	PCR	Outbreak
Bangladesh	[28]	Cattle	Epithelium	12	10	RT‐PCR	Endemic
Bangladesh	[29]	Cattle	Vesicle	31	30	ELISA and RT‐PCR	Outbreak
Bhutan	[30]	Cattle, buffalo, goat, and pig	Blood	1909	287	ELISA	Endemic
Cambodia	[31]	Cattle and buffalo	Blood	972	779	ELISA	Endemic
Cambodia	[32]	Cattle and pig	Blood	2238	372	ELISA	Endemic
China	[33]	Cattle	Tissue and vesicular fluid	1648	369	RT‐PCR	Endemic
China	[34]	Cattle	Swab, fluid, blood, and tissue	143	85	RT‐PCR	Endemic
India	[35]	Cattle	Blood and oropharyngeal fluid	20	3	RT‐PCR	Outbreak
India	[36]	Cattle	Oropharyngeal fluid	36	4	RT‐PCR	Outbreak
India	[37]	Gayal	Epithelium	15	12	ELISA	Outbreak
India	[38]	Sheep and goat	Blood	129	85	RT‐PCR	Outbreak
India	[39]	Goat	Blood	617	235	ELISA	Endemic
India	[40]	Sheep and goat	Blood	8442	1446	ELISA	Outbreak
India	[41]	Sheep and goat	Epithelium and vesicular fluid	500	166	ELISA	Outbreak
India	[42]	Cattle and buffalo	Blood	8877	3034	ELISA	Outbreak
India	[43]	Cattle and buffalo	Blood	400	111	ELISA	Outbreak
Indonesia	[44]	Cattle	Swab and vesicular fluid	26	12	RT‐PCR	Endemic
Indonesia	[45]	Cattle	Blood	32	21	ELISA	Endemic
Iran	[46]	Cattle, sheep, and goat	Epithelium and tissue	71	24	RT‐PCR	Outbreak
Iran	[47]	Cattle	Epithelium	50	10	RT‐PCR	Outbreak
Iran	[48]	Cattle	Tissue	255	96	RT‐PCR	Endemic
Iran	[49]	Cattle, sheep, and goat	Epithelium and vesicular fluid	128	86	ELISA	Endemic
Iraq	[50]	Buffalo	Tissue	70	61	RT‐PCR	Outbreak
Iraq	[51]	Cattle	Blood	84	29	ELISA	Endemic
Iraq	[52]	Cattle	Saliva and vesicular fluid	73	55	RT‐PCR	Endemic
Iraq	[53]	Cattle	Vesicular fluid	73	55	RT‐PCR	Outbreak
Laos	[54]	Goat	Blood	591	77	ELISA	Endemic
Laos	[55]	Cattle, buffalo, and pig	Blood	1280	292	ELISA	Endemic
Laos	[56]	Cattle and goat	Blood	972	340	ELISA	Endemic
Laos	[57]	Cattle and buffalo	Blood	2663	1225	ELISA	Endemic
Laos	[58]	Cattle and buffalo	Blood	4247	2145	ELISA	Endemic
Laos	[59]	Pig	Blood	647	119	ELISA	Endemic
Laos	[60]	Cattle, buffalo, and goat	Blood and swab	621	268	ELISA and RT‐PCR	Endemic
Myanmar	[61]	Cattle and buffalo	Swab	130	68	ELISA and PCR	Endemic
Nepal	[62]	Cattle, buffalo, sheep, goat, and pig	Blood, swab, and tissue	3216	474	ELISA and RT‐PCR	Endemic
Nepal	[63]	Cattle and goat	Blood	650	112	ELISA	Endemic
Oman	[64]	Cattle, sheep, and goat	Blood	5807	1187	ELISA	Endemic
Pakistan	[65]	Sheep and goat	Blood	1478	337	ELISA	Outbreak
Pakistan	[66]	Cattle, buffalo, sheep, and goat	Swab	109	77	ELISA	Outbreak
Pakistan	[67]	Cattle, buffalo, sheep, and goat	Blood	376	61	ELISA	Endemic
Pakistan	[68]	Cattle and buffalo	Tissue	200	114	ELISA	Endemic
Pakistan	[69]	Sheep	Blood and tissue	95	32	ELISA and RT‐PCR	Outbreak
Pakistan	[70]	Cattle and buffalo	Blood	2511	247	ELISA	Endemic
Pakistan	[71]	Cattle and buffalo	Swab	324	109	RT‐PCR	Endemic
Pakistan	[72]	Cattle and buffalo	Blood and epithelium	125	89	RT‐PCR	Outbreak
Pakistan	[73]	Buffalo	Oropharyngeal fluid	300	180	RT‐PCR	Endemic
Pakistan	[74]	Cattle, buffalo, and goat	Tissue and secretion	425	274	RT‐PCR	Outbreak
Pakistan	[75]	Cattle and buffalo	Epithelium, tissue, and vesicular fluid	58	41	RT‐PCR	Endemic
Pakistan	[76]	Cattle, buffalo, sheep, and goat	Epithelium and vesicular fluid	233	179	ELISA and RT‐PCR	Outbreak
Saudi Arabia	[77]	Sheep	Blood	50	38	ELISA	Endemic
Saudi Arabia	[78]	Sheep and goat	Blood	162	6	ELISA	Endemic
Saudi Arabia	[79]	Cattle, camel, sheep, and goat	Blood	841	147	ELISA	Endemic
South Korea	[80]	Cattle and buffalo	Tissue and saliva	55	51	RT‒PCR	Outbreak
South Korea	[81]	Pig	Tissue, saliva, and vesicle	40	3	ELISA and RT‐PCR	Outbreak
South Korea	[82]	Pig	Blood, oropharyngeal fluid, and tissue	69	39	RT‐PCR	Outbreak
Türkiye	[83]	Goat	Blood	368	12	ELISA	Endemic
Türkiye	[84]	Cattle and sheep	Blood	190	13	ELISA and	Endemic
UAE	[85]	Oryx	Blood	131	90	ELISA and RT‐PCR	Outbreak
Vietnam	[86]	Cattle and buffalo	Epithelium and blood	1446	323	ELISA and RT‐PCR	Outbreak
Vietnam	[87]	Cattle and buffalo	Oropharyngeal fluid	5045	446	RT‐PCR	Outbreak
Vietnam	[88]	Pig	Epithelium	378	351	RT‐PCR	Outbreak

### 2.5. Bias Assessment and Quality Evaluation of the Studies

The reporting quality and potential selection bias of the studies included in this meta‐analysis were assessed using a standardized quality appraisal checklist [89]. This tool comprised seven predefined criteria, with each item scored dichotomously (1 = yes; 0 = no), and an overall mean quality score calculated for each study. Based on the total scores, studies were stratified into three quality categories: low quality (scores 0–3), indicating a high risk of bias; moderate quality (scores 4–5), indicating a moderate risk of bias; and high quality (scores 6–7), indicating a low risk of bias (S2 and S3) [89]. To assess the robustness of the results, sensitivity analyses were performed to evaluate the effects of excluding studies assessed as high or moderate risk of bias. This systematic approach to quality appraisal strengthens the reliability of the review by explicitly identifying and accounting for potential sources of bias within the included studies.

### 2.6. Statistical Analysis

The extracted data were transcribed and systematically entered into a Microsoft Excel spreadsheet [90]. A random‐effects meta‐analysis was applied to estimate the pooled prevalence of FMD with corresponding 95% confidence intervals (CIs) [91, 92]. Heterogeneity was quantified using the *I* ^2^ statistic and interpreted as low (25%), moderate (50%), or high (75%), with values of 0% indicating no observed heterogeneity [93]. Owing to the substantial heterogeneity across the included studies, a random‐effects model was selected for all summary estimates. Subgroup analyses were conducted according to country, diagnostic method, host species, year of study, and disease occurrence. In addition, random‐effects meta‐regression was performed to evaluate temporal trends in FMD prevalence across different species. Publication bias was assessed visually using funnel plots and statistically using Egger’s regression test to detect funnel plot asymmetry and small‐study effects [94]. A *p* value of < 0.05 in Egger’s test was considered indicative of statistically significant publication bias. Sensitivity analysis was undertaken by sequentially omitting individual studies to examine the robustness and stability of the pooled prevalence estimates. All meta‐analytical procedures were performed using Stata version 18.0 (College Station, TX, USA). Spatial visualization was carried out using R software (version 4.4.2), employing relevant packages such as spdep and ggplot for map generation [95, 96]. This systematic review and meta‐analysis were conducted in accordance with the PRISMA guidelines [97].

## 3. Results

### 3.1. Search Results and Eligible Studies

**FIGURE 1:**
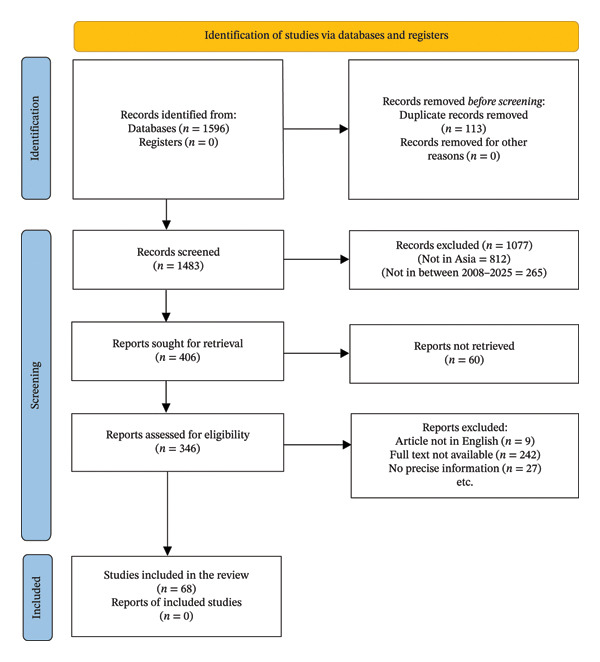
PRISMA flow diagram for the inclusion of eligible articles in the meta‐analysis.

Figure 1 illustrates the search results and the number of eligible studies for this review and meta‐analysis. A systematic review identified 1596 records from databases, removing 113 duplicates. Of the 1483 remaining records, 406 reports were retrieved, with 60 not retrieved. After screening, 346 reports were assessed for eligibility; 242 lacked full text, 27 had unavailable prevalence data, and nine (9) were non‐English. Ultimately, 68 new studies were included. A total of 68 articles meeting the eligibility criteria were included in the final analysis [9–11, 26, 29, 30, 39, 48, 71, 74, 75] [27, 28, 31, 36–38, 40–42, 52, 65, 69, 70, 72, 73, 77, 78, 81, 83, 86] [24, 25, 34, 35, 43, 47, 49, 53, 54, 59, 61, 64, 66, 67, 79, 82, 84, 85, 87, 88] [32, 33, 44–46, 50, 51, 55–58, 60, 62, 63, 68, 76, 80].

### 3.2. Descriptive Characteristics of the Included Studies

Among the 1596 studies identified through the literature search, 68 studies were included on the basis of the criteria of this systematic review and meta‐analysis, covering the prevalence of FMD in 19 Asian countries between 2008 and 2025. The diagnosis of FMDV was conducted by different laboratory techniques, including PCR, RT‐PCR, ELISA, and a combination of ELISA and PCR or RT‐PCR. Additionally, this study included several species, such as cattle, buffalo, sheep, goats, pigs, horses, gayal, and Oryx, in which FMD was detected. Moreover, the data were divided into different disease occurrences, including endemic and outbreak. Furthermore, various types of samples, such as epithelium, blood, tissue, swabs, saliva, and different types of fluid, were included in this study. Those studies mentioned the serotypes, which were also recorded in this study (Table 1). In these studies, a total of 71,773 samples were recorded, and 21,884 samples were identified as FMDV‐positive. Moreover, the highest and lowest sample sizes were 8877 and 12, respectively. Prevalence rates varied significantly across regions. However, the highest prevalence of FMD was 96.77% (95% CI: 86.67%–97.47%) in Bangladesh [29], and the lowest rate was 3.26% (95% CI: 1.66%–5.35%) in Türkiye [83].

### 3.3. Continents and Countries

This study includes (*n* = 68) articles, all sourced from Asia and representing 19 countries within the region: Afghanistan (*n* = 3) [24–26], Bangladesh (*n* = 6) [9–11, 27–29], Bhutan (*n* = 1) [30], Cambodia (*n* = 2) [31, 32], China (*n* = 2) [33, 34], India (*n* = 9) [35–43], Indonesia (*n* = 2) [44, 45], Iraq (*n* = 4) [50–53], Iran (*n* = 4) [46–49], Laos (*n* = 7) [54–60], Myanmar (*n* = 1) [61], Nepal (*n* = 2) [62, 63], Oman (*n* = 1) [64], Pakistan (*n* = 12) [65–76], Saudi Arabia (*n* = 3) [77–79], South Korea (*n* = 3) [80–82], Türkiye (*n* = 2) [83, 84], UAE (*n* = 1) [85], and Vietnam (*n* = 3) [86–88].

### 3.4. Meta‐Analysis of FMD in Asia

**FIGURE 2:**
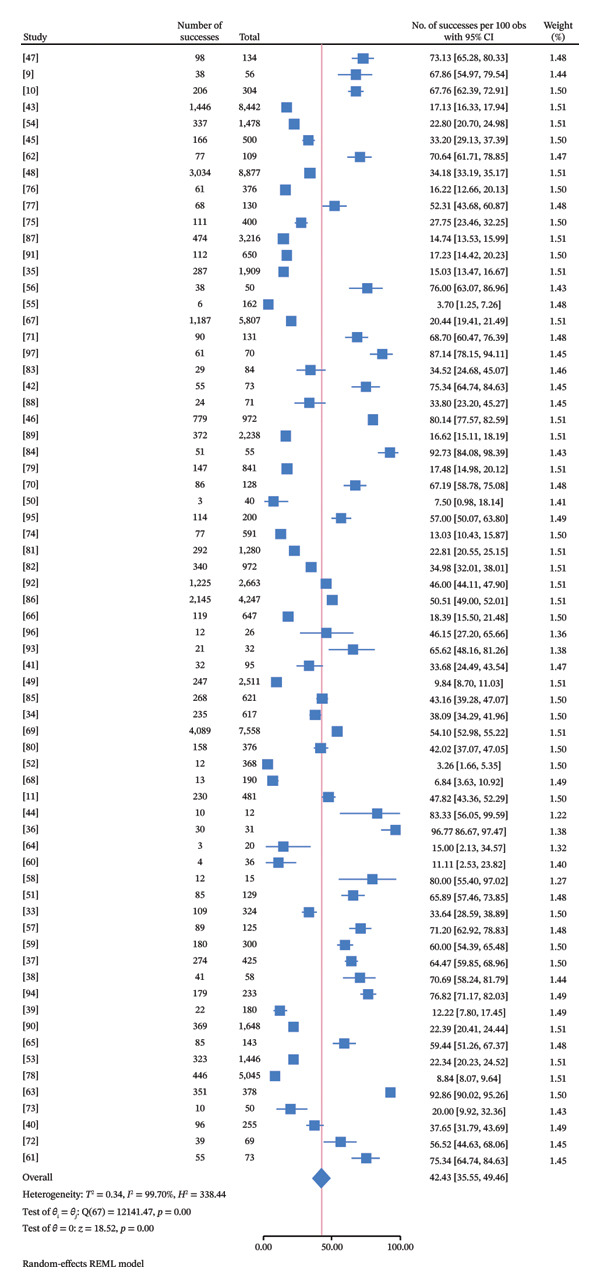
Forest plot of the meta‐analysis of foot and mouth disease (FMD) in Asia.

The random‐effects meta‐analysis revealed that the pooled prevalence of FMD was 42.43% (95% CI: 35.55%–49.46%, *τ* ^2^ = 0.34, *I* ^2^ = 99.70, *p* < 0.0001). The forest plot illustrates the pooled prevalence of FMD in Asia (Figure 2). The individual study weights showed minimal variation from 1.22% to 1.51%.

### 3.5. Subgroup Meta‐Analysis

**FIGURE 3:**
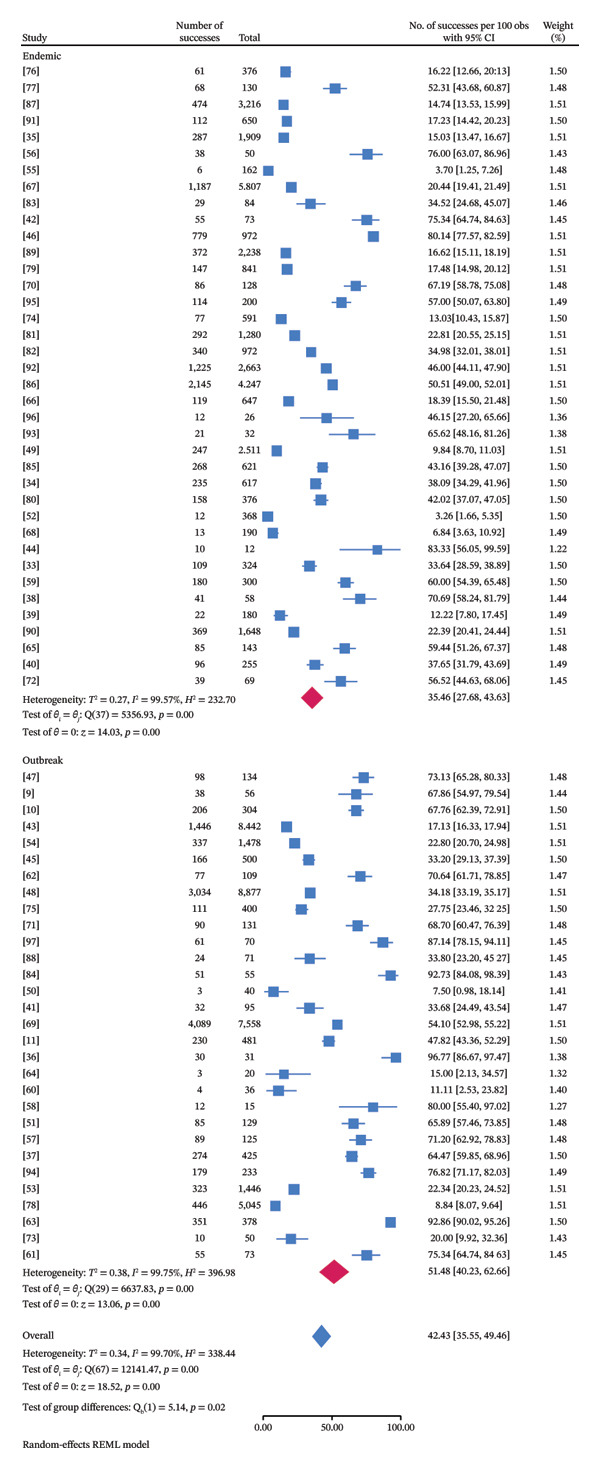
Subgroup meta‐analysis based on disease occurrence.

**FIGURE 4:**
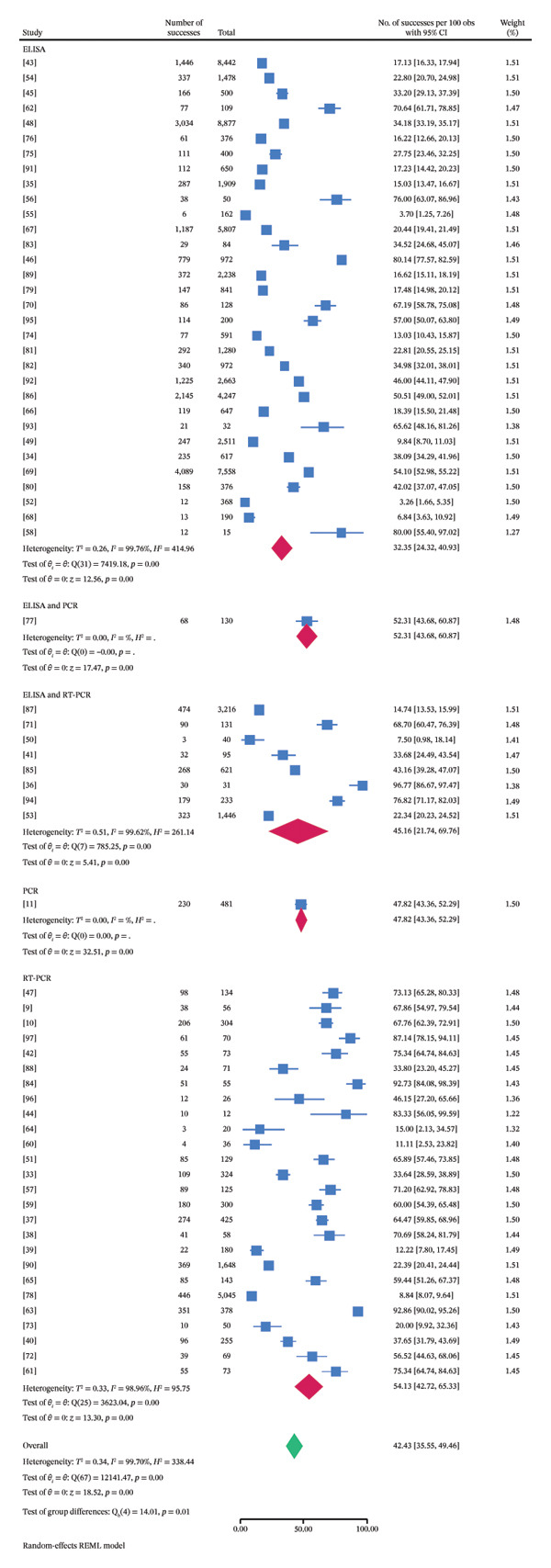
Subgroup meta‐analysis based on different detection methods.

**FIGURE 5:**
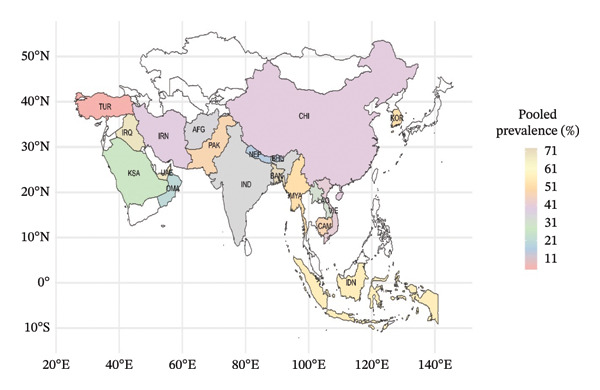
Countries with pooled prevalence of FMD (Table 4) in different Asian countries (map was created using R programming).

Subgroup meta‐analyses were conducted on the basis of variables such as sample type, species, country in Asia, diagnostic technique, disease occurrence pattern, and publication year (Tables 2–5, Figures 3 and 4). Species‐specific pooled prevalence estimates of FMD were derived through meta‐analysis, with 80% (95% CI: 55.40–97.02) being the highest in Gayal (a single study), 74.30% (95% CI: 44.66%–95.20%) in buffalo and 6.84% (95% CI: 3.63%–10.92%) being the lowest in goats (15.62%, 95% CI: 1.67%–39.64%). Among the Asian countries included in the analysis, the highest pooled prevalence of FMD was observed in Bangladesh (72.86%; 95% CI: 57.57%–85.90%; *τ* ^2^: 0.13; *I* ^2^: 94.83; *H* ^2^ = 19.33; *p* < 0.0001), whereas the lowest estimate was reported in Türkiye (4.73%; 95% CI: 1.80%–8.87%; *τ* ^2^: 0.01; *I* ^2^: 71.77; *H* ^2^ = 3.54; *p* < 0.0001) (Figure 5). On the basis of the different types of samples used to diagnose FMD, subgroup analysis revealed that vesicles had the highest pooled prevalence of FMD (96.77%; 95% CI: 86.67%–100%), whereas the lowest prevalence (7.50%; 95% CI: 0.98%–18.14%) was observed in tissues, saliva, and vesicles. Prevalence estimates varied significantly by diagnostic procedure, with higher rates detected through RT‐PCR (54.13%; 95% CI: 42.72–65.33; *τ* ^2^ = 0.33; *I* ^2^ = 98.96; *H* ^2^ = 95.75; *p* < 0.0001) and ELISA and PCR (52.31%; 95% CI: 43.68%–60.87%) than via PCR (47.82%; 95% CI: 43.36%–52.29%) and ELISA (32.35%; 95% CI: 24.32%–40.93%; *τ* ^2^ = 0.26; *I* ^2^ = 99.76; *H* ^2^ = 414.96; *p* < 0.0001). Temporal trends indicated the highest pooled prevalence of FMD in 2025 (87.14%; 95% CI: 78.15%–94.11%), whereas the lowest estimate was observed in 2017 (21.19%; 95% CI: 0.45–58.87; *τ* ^2^ 0.64; *I* ^2^ = 99.49; *H* ^2^ = 195.87; *p* < 0.0001). In terms of disease occurrence, the pooled estimates were greater during outbreak conditions (51.48%, 95% CI: 40.23–62.66; *τ* ^2^ 0.38; *I* ^2^ = 99.75; *H* ^2^ = 396.98; *p* < 0.0001) than during endemic conditions (35.46%, 95% CI: 27.68–43.63; *τ* ^2^ 0.27; *I* ^2^ = 99.57; *H* ^2^ = 232.70; *p* < 0.0001).

**TABLE 2:** Subgroup meta‐analysis by year.

Variable	Total sample	FMD positive	Pooled estimate (%)	95% CI	*τ* ^2^	*I* ^2^ %	*p* value	*H* ^2^
2008	324	109	33.64	28.59–38.89	0.00	—^∗^	—	—
2010	648	265	71.62	9.76–100.00	0.94	98.25	< 0.0001	57.12
2011	2572	624	38.40	10.75–70.95	0.45	99.38	< 0.0001	162.05
2012	255	96	37.65	31.79–43.69	0.00	—	—	—
2013	8537	1478	24.31	10.13–42.16	0.07	92.98	< 0.0001	14.25
2014	73	55	75.34	64.74–84.63	0.00	—	—	—
2015	3026	1319	47.36	21.17–74.32	0.45	99.43	< 0.0001	175.62
2016	11,651	3464	44.07	15.71–74.73	0.41	99.83	< 0.0001	600.84
2017	2058	393	21.19	0.45–58.87	0.64	99.49	< 0.0001	195.87
2018	1503	1062	61.58	43.84–77.91	0.30	97.68	< 0.0001	43.14
2019	14,202	5408	23.24	7.27–44.72	0.22	99.82	< 0.0001	562.76
2020	1745	474	37.04	19.45–56.58	0.27	98.39	< 0.0001	61.99
2021	9265	1719	30.26	18.83–43.08	0.13	99.23	< 0.0001	130.2
2022	4386	2225	61.35	24.22–92.19	0.44	98.38	< 0.0001	61.67
2023	7654	1469	25.98	15.00–38.73	0.09	99.19	< 0.0001	124.12
2024	3804	1663	50.93	32.93–68.81	0.19	98.58	< 0.0001	70.52
2025	70	61	87.14	78.15–94.11	0.00	—	—	—

^∗^Not calculated because only one study was available on this topic.

**TABLE 3:** Subgroup meta‐analysis based on species.

Variable	Total sample	FMD positive	Pooled estimate (%)	95% CI	*τ* ^2^	*I* ^2^ %	*p* value	*H* ^2^
Buffalo	370	241	74.30	44.66–95.20	0.18	95.46	< 0.0001	22.05
Cattle	3049	1073	51.60	37.61–65.47	0.30	97.62	< 0.0001	42.08
Gayal	15	12	80.00	55.40–97.02	0.00	—^∗^	—	—
Goat	1576	324	15.62	1.67–39.64	0.23	99.17	< 0.0001	120.50
Oryx	131	90	68.70	60.47–76.39	0.00	—	—	—
Pig	1134	512	43.83	7.32–84.85	0.80	99.41	< 0.0001	170.76
Sheep	145	70	54.95	15.45–91.11	0.36	95.94	< 0.0001	24.66
Sheep and goat	10,771	2040	26.03	8.55–48.82	0.29	99.64	< 0.0001	278.35
Cattle and buffalo	27,233	8804	43.57	29.30–58.41	0.34	99.81	< 0.0001	536.37
Cattle and goat	1622	452	25.63	10.51–44.60	0.08	98.46	< 0.0001	65.06
Cattle and pig	2542	578	40.76	2.37–88.77	0.59	99.69	< 0.0001	319.91
Cattle and sheep	190	13	6.84	3.63–10.92	0.00	—	—	—
Cattle, buffalo, and goat	1046	542	53.86	33.09–73.95	0.09	97.86	< 0.0001	46.70
Cattle, buffalo, and pig	1761	522	34.72	13.04–60.44	0.14	98.99	< 0.0001	98.59
Cattle, buffalo, and sheep	7558	4089	54.10	52.98–55.22	0.00	—	—	—
Cattle, sheep, and goat	6006	1297	39.67	14.62–68.01	0.25	97.98	< 0.0001	49.43
Cattle, buffalo, sheep, and goat	718	317	54.02	16.43–89.10	0.51	99.09	< 0.0001	110.37
Cattle, camel, sheep, and goat	841	147	17.48	14.98–20.12	0.00	—	—	—
Cattle, buffalo, goat, and pig	1909	287	15.03	13.47–16.67	0.00	—	—	—
Cattle, buffalo, sheep, goat, and pig	3216	474	14.74	13.53–15.99	0.00	—	—	—

^∗^Not calculated because only one study was available on this topic.

**TABLE 4:** Subgroup meta‐analysis based on different countries in Asia.

Variable	Total sample	FMD positive	Pooled estimate (%)	95% CI	*τ* ^2^	*I* ^2^ %	*H* ^2^	*p* value
Afghanistan	8114	4269	34.91	12.30–61.91	0.23	99.18	122.02	< 0.0001
Bangladesh	1018	612	72.86	57.57–85.90	0.13	94.83	19.33	< 0.0001
Bhutan	1909	287	15.03	13.47–16.67	0.00	—^∗^	—	—
Cambodia	3210	1151	47.88	0.76–98.31	0.95	99.92	1285.24	< 0.0001
China	1791	454	39.93	8.89–76.47	0.30	98.73	78.92	< 0.0001
India	19,036	5096	34.12	20.95–48.61	0.18	99.61	254.62	< 0.0001
Indonesia	58	33	56.50	37.30–74.80	0.04	53.11	2.13	< 0.0001
Iran	504	216	39.68	21.25–59.72	0.15	94.44	17.97	< 0.0001
Iraq	300	200	69.17	45.37–88.64	0.23	94.46	18.04	< 0.0001
Laos	11,021	4466	31.88	21.25–43.57	0.11	99.33	148.54	< 0.0001
Myanmar	130	68	52.31	43.68–60.87	0.00	—	—	—
Nepal	3866	586	15.64	13.33–18.09	0.00	61.26	2.58	< 0.0001
Oman	5807	1187	20.44	19.41–21.49	0.00	—	—	—
Pakistan	6234	1740	47.96	33.46–62.63	0.27	99.14	116.34	< 0.0001
Saudi Arabia	1053	191	27.90	0.08–75.81	0.76	99.26	135.54	< 0.0001
South Korea	164	93	52.61	5.42–96.85	0.96	98.10	52.57	< 0.0001
Türkiye	558	25	4.73	1.80–8.87	0.01	71.77	3.54	< 0.0001
UAE	131	90	68.70	60.47–76.39	0.00	—	—	—
Vietnam	6869	1120	41.21	0.92–92.64	1.12	99.94	158.28	< 0.0001

^∗^Not calculated because only one study was available on this topic.

**TABLE 5:** Subgroup meta‐analysis based on various types of samples.

Variable	Total sample	FMD positive	Pooled estimate (%)	95% CI	*τ* ^2^	*I* ^2^ %	*p* value	*H* ^2^
Blood	54,469	17,009	29.02	21.16–37.57	0.23	99.77	< 0.0001	428.85
Blood and epithelium	1571	412	46.02	5.74–89.96	0.52	99.17	< 0.0001	120.14
Blood and oropharyngeal fluid	20	3	15	2.13–34.57	0.00	—^∗^	—	—
Blood and swab	621	268	43.16	39.28–47.07	0.00	—	—	—
Blood and tissue	95	32	33.68	24.49–43.54	0.00	—	—	—
Blood, oropharyngeal fluid, and tissue	69	39	56.52	44.63–68.06	0.00	—	—	—
Blood, swabs, and tissue	3216	474	14.74	13.53–15.99	0.00	—	—	—
Epithelium	1559	1040	67.05	51.51–80.95	0.21	96.95	< 0.0001	32.79
Epithelium and tissue	71	24	33.80	23.20–45.27	0.00	—	—	—
Epithelium and vesicular fluid	861	431	59.35	32.69–83.37	0.22	98.20	< 0.0001	55.44
Epithelium, tissue, and vesicular fluid	58	41	70.69	58.24–81.79	0.00	—	—	—
Oropharyngeal fluid	5381	630	24.16	1.70–60.10	0.42	99.25	< 0.0001	133.15
Saliva and vesicular fluid	73	55	75.34	64.74–84.63	0.00	—	—	—
Swab	743	276	40.93	17.11–67.24	0.29	98.03	< 0.0001	50.89
Swab and vesicular fluid	26	12	46.15	27.20–65.66	0.00	—	—	—
Swab, fluid, blood, and tissue	143	85	59.44	51.26–67.37	0.00	—	—	—
Tissue	525	271	61.54	31.42–87.51	0.28	97.81	< 0.0001	45.61
Tissue and saliva	55	51	92.73	84.08–98.39	0.00	—	—	—
Tissue and secretion	425	274	64.47	59.85–68.96	0.00	—	—	—
Tissue and vesicular fluid	1648	369	22.39	20.41–24.44	0.00	—	—	—
Tissue, saliva, and vesicles	40	3	7.50	0.98–18.14	0.00	—	—	—
Vesicle	31	30	96.77	86.67–100	0.00	—	—	—
Vesicular fluid	73	55	75.34	64.74–84.63	0.00	—	—	—

^∗^Not calculated because only one study was available on this topic.

### 3.6. Meta‐Regression

**FIGURE 6:**
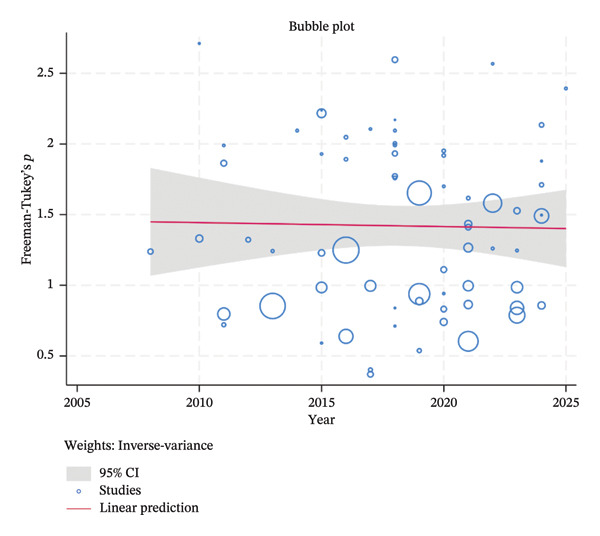
Meta‐regression of the prevalence of FMD in different animals and year of study.

The meta‐regression analysis indicated no statistically significant linear relationship between the year of publication and the Freeman–Tukey transformed proportion (*p* = 0.874). The coefficient for the year (0.002785) suggests a negligible decrease in the transformed proportion per year, but the 95% CI (−0.0373146, 0.0317445) includes zero, confirming the lack of significance. The model explained 0% of the heterogeneity (*R*‐squared = 0.00%), and significant residual heterogeneity remained (*I* ^2^ = 99.70%, *p* < 0.0001), indicating that other factors likely contributed to the variability across studies. The bubble plot visually confirms this finding, displaying a flat, linear prediction line with a wide 95% CI, indicating high heterogeneity and no clear trend over time (Figure 6).

### 3.7. Publication Bias

**FIGURE 7:**
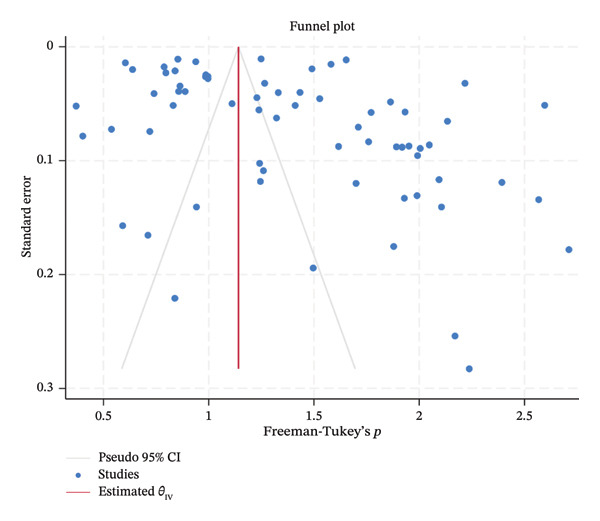
A funnel plot illustrating publication bias in different studies.

To assess the presence of publication bias in our study, we conducted Egger’s regression test for small‐study effects using a random‐effects model and a funnel plot analysis. Egger’s test revealed a statistically significant result (*β* = 3.97, SE = 1.148, *z* = 3.46, *p* = 0.0005), indicating the presence of small‐study effects and potential publication bias in the included studies. In addition, the funnel plot revealed an asymmetric distribution of studies around the pooled effect size (Freeman–Tukey’s *p*), particularly with a greater spread on the right side of the plot. This asymmetry further supports the presence of publication bias (Figure 7).

### 3.8. Sensitivity Analysis

**FIGURE 8:**
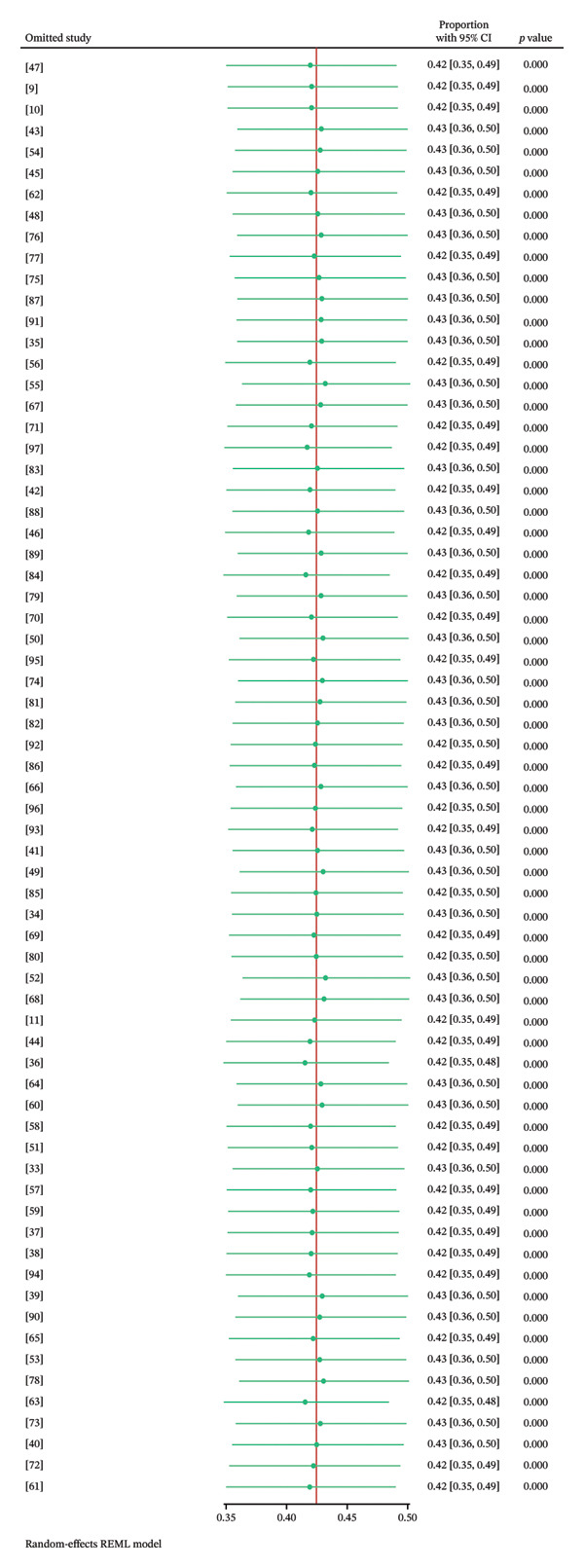
Sensitivity analysis of the prevalence of FMD in Asia from 2012 to 2025.

To assess the robustness of the meta‐analysis results, a leave‐one‐out sensitivity analysis was conducted through the random‐effects REML model (Figure 8). Each of the 68 studies was systematically excluded one at a time, and the pooled prevalence was recalculated to assess the influence of individual studies on the overall estimate. The analysis included 68 studies, with individual study proportions ranging from 35% to 49%. The pooled proportion across all studies was 42% (95% CI: 35%–49%). When each study was excluded one at a time, the recalculated pooled proportions remained stable, ranging from 41% to 43%, with 95% CIs consistently overlapping the overall estimate (35%–49%). Remarkably, the exclusion of Giasuddin et al. [27] resulted in the lowest pooled proportion of 42% (95% CI: 35%–48%), whereas removing Lim et al. [80] yielded the highest at 43% (95% CI: 36%–50%). These findings showed that no single study disproportionately influenced the overall pooled estimate, confirming the stability of the meta‐analysis results.

### 3.9. Serotypes of FMD

Table 6 shows the distribution of FMDV serotypes across various countries in Asia, highlighting distinct geographical and species‐specific patterns of occurrence. The study demonstrated that FMD serotype O was the most prevalent in the Asian region and was detected in all the countries included in the analysis. In Nepal, a diverse range of animal species, including cattle, buffalo, sheep, goats, and pigs, were affected by serotypes O and A. Serotypes O, A, and Asia 1 were primarily observed in cattle and buffalo populations in countries such as Bangladesh, India, Pakistan, Afghanistan, China, Cambodia, Laos, South Korea, Georgia, and Tajikistan. Less commonly affected hosts, including sheep and goats, were reported with serotypes O, A, and Asia 1 in India, Saudi Arabia, Laos, Pakistan, Türkiye, and Georgia. Remarkably, wildlife species such as the gayal in India and the oryx in the UAE tested positive for serotype O. Additionally, pigs in Bangladesh and Laos were found to carry serotypes A, O, and Asia 1, whereas horses in Saudi Arabia presented infections with serotypes O and A. An exclusive case was documented in Iraq, in which buffalo were infected with the less common SAT 2 serotype. Moreover, serotype O is prevalent in almost every country in Asia, except Iraq.

**TABLE 6:** Distribution of FMDV serotypes.

Country	Species	Serotypes	References
Bangladesh	Cattle, buffalo, and pig	A, O, Asia 1	[9, 10, 11, 27, 28, 29, 98]

India	Sheep and goat	A, O, Asia 1	[38, 39, 40, 41]
	Cattle and buffalo	A, O, Asia 1	[35, 36, 41, 42, 99]

India	Gayal	O	[37]

Nepal	Cattle, buffalo, sheep, goat, and pig	O, A	[62, 100]

Iraq	Buffalo	SAT 2	[50]
	Cattle	A	[53]

Cambodia	Cattle and buffalo	A, O, Asia 1	[31]

South Korea	Cattle and buffalo	O, A, Asia 1	[80–82]

Saudi Arabia	Cattle, sheep, and horse	O, A	[77, 101]

Iran	Cattle, sheep, and goat	A, O, Asia 1	[46–49]

Laos	Cattle, buffalo, goat, and pig	O, A, Asia 1	[54–56, 59]

Indonesia	Cattle	O	[44]

Afghanistan	Cattle, buffalo, and sheep	O, A, Asia 1	[24, 26]

Türkiye	Cattle and sheep	O, A	[84]

Georgia	Cattle, buffalo, sheep, and goat	O, A, Asia 1	[102]

Pakistan	Cattle, buffalo, sheep, and goat	A, O, Asia 1	[26, 66, 69, 71–76]

Tajikistan	Cattle and buffalo	O, A, Asia 1	[26]

China	Cattle	O, A, Asia 1	[33, 34]

Vietnam	Cattle, buffalo, and pig	O, A	[86–88]

Myanmar	Cattle	O	[88]

UAE	Cattle, sheep, goat, and oryx	O	[85, 103]

## 4. Discussion

To our knowledge, this meta‐analysis represents the first study to pool estimates of FMD prevalence in Asia. This study plays a crucial role in understanding FMD prevalence in Asia, reinforcing the need for effective disease management policies. The data were gathered from a systematic review of published research articles spanning the years 2008–2025. This review encompasses livestock (cattle, buffalo, sheep, goat, pig, and horse) and wild animals, including gayal and oryx. This review and meta‐analysis included 68 studies, as studies reporting FMD prevalence were limited by factors such as publication in English, availability of full texts, and restriction to Asian countries. The articles were obtained from 19 out of 47 Asian countries, with the highest number from Pakistan, India, Laos, Bangladesh, Iran, and Iraq. The studies employed various diagnostic tests for FMD detection, including ELISA, PCR, and RT‐PCR. Among the FMD virus serotypes, serotype O was the most prevalent, followed by serotypes A, Asia 1, and SAT 2. The majority of the articles described study designs such as cross‐sectional studies, field investigations, longitudinal studies, retrospective analyses, serological studies, and surveys. The limited number of studies from certain countries may have influenced the overall findings. In some regions, the absence of FMD or its low‐priority status could explain the lack of published research. Additionally, outbreaks may be localized and quickly occurring, reducing the perceived need for extensive study. Inadequate diagnostic capacity in some countries also contributes to the scarcity of research, which is consistent with previous findings [2].

The pooled prevalence estimate of FMD in wild and livestock animals was 42.43% (95% CI: 35.55%–49.46%), which is higher than that reported in other studies conducted in African countries, such as 21.39% in Ethiopia (East African country) [5] and 16% in Africa [3]. The possible explanation for the elevated FMD rate could be attributed to factors such as high livestock density, informal trade, and livestock migration. Comparing the estimates of this study with findings from individual studies is often challenging. This study revealed a relatively high prevalence of FMD, which poses a significant threat because of its impact on live animals, products, and byproducts, especially since FMD is classified as a transboundary List A disease. Additionally, it adversely affects farmers’ livelihoods and the national economy, particularly the livestock sector as a whole [104]. The presence of multiple FMDV serotypes in the region reduces vaccine effectiveness, making it challenging to develop specific and long‐lasting immunity. Moreover, serotype O viruses in Asia exhibit low antigenic matching with current vaccine strains, particularly the Cathay topotype. This makes vaccine selection and disease control challenging [105].

FMDV is classified into seven (7) different serotypes (O, A, C, Asia 1, SAT 1–3) on the basis of antigenic differences. In Asian countries, FMD is endemic and affected by three (3) main serotypes (O, A, and Asia 1). However, SAT 2 was also identified in Iraq from Buffalo [50]. The FMDV serotype SAT2 is the most widely distributed and frequently associated with livestock outbreaks in Africa, as supported by [106]. Its long‐standing presence, dating back to the 15th–16th centuries, may explain its dominance. Historical records trace FMD in Africa to the 1780s [107]. In South Asia, India reports serotype O as the dominant strain responsible for approximately 92% of outbreaks, followed by Asia‐1 (5%) and A (3%) [108]. Similarly, Bangladesh (82% O serotype, 11% A serotype) and Nepal (98% O serotype, 2% A serotype) followed the same trend [11, 62]. Serotype O is widespread in Vietnam, whereas serotype A is confined to the Northeast, Central, and Southern regions of Vietnam. Interestingly, serotype Asia‐1 has not been detected since 2007 [109]. The movement of livestock, particularly cattle and buffalo, is a significant factor in the spread of FMD. This is evident in countries such as India and Bangladesh, where animal movement contributes to disease transmission [11, 108]. Moreover, airborne transmission of FMDV, although less common than direct contact, poses a serious risk under favorable conditions by enabling the virus to spread beyond quarantine zones. Understanding aerosol generation, the viral load, travel distance, and environmental survival is essential for effective control. As much of the existing data are outdated, modern tools and modeling approaches are needed to enhance current knowledge and strengthen outbreak prevention strategies [110].

In the subgroup analysis, the highest pooled prevalence of FMD in buffaloes (74.30%) was greater than that in other species, such as cattle (51.60%), sheep (54.95%), goats (15.62%), and pigs (43.83%). Research has shown that African buffaloes are systemically affected by FMDV and act as key wildlife reservoirs for the virus [111, 112]. Although viral persistence in buffalo has been observed, some studies suggest that it may not be common, and the exact transmission mechanism remains unclear [111, 113]. Additionally, geographic features such as rivers have been found to influence FMDV circulation in buffalo populations [113]. In contrast, cattle are the most studied animals because of their socioeconomic value and primary livelihood sources. The lower pooled estimates were observed in goats aligned with those of Wolf et al. [114], who noted that these species often show mild or no clinical signs of FMD.

In this study, the pooled prevalence of FMD according to RT‒PCR (54.13%) was greater than that according to other diagnostic techniques, such as PCR (47.82%) and ELISA (32.35%). Only a few studies have utilized PCR techniques, which is consistent with the findings of Howson et al. [115], highlighting the preference for PCR in endemic settings because of its simplicity, high sensitivity, and rapid detection. In our study, the pooled estimate of FMD in outbreak settings (51.48%) was compared with that in endemic settings (35.46%). The prevalence of FMD differs significantly between outbreak and endemic settings, driven by factors such as disease management, animal movement, and environmental conditions. Outbreak settings are characterized by high morbidity and rapid spread, whereas endemic settings exhibit persistent, low‐level circulation sustained by subclinical infections and inadequate control measures [116, 117].

Among the 19 Asian countries, Bangladesh (72.8) had the highest pooled prevalence (Figure 5 and Table 4), and the lowest rate was in Türkiye (4.73), followed by Bhutan (15.03) and Nepal (15.60). The high FMD prevalence in Bangladesh is influenced by dense livestock populations, traditional farming practices, climatic conditions, and cross‐border animal movements [11, 14]. Türkiye FMD prevalence varies by region. The Thrace region has been declared FMD‐free, whereas the East Anatolian and Southeast Anatolian regions report higher FMD incidence due to factors such as illegal animal movements and lower vaccination coverage. Mass vaccination and quarantine measures have been implemented, which have been effective in certain regions, but challenges remain in areas with less stringent enforcement. Nepal has implemented various control measures that have contributed to a comparatively lower prevalence. The country’s varied topography, including hills and mountainous regions, acts as a natural barrier, limiting the movement of animals and thereby reducing the spread of FMD. The introduction and scaling up of vaccination campaigns since 2010 have also played a significant role in controlling FMD outbreaks [62].

### 4.1. Limitations

This study has several limitations including the following: (i) out of 47 Asian countries, this study was based on 19 countries, which may limit the representativeness of the pooled estimates and introduce geographical bias toward countries with more developed surveillance and reporting systems; (ii) the review excluded non‐English articles, unpublished articles, experimental trials, and case reports, which may have led to publication and language bias, potentially underestimating or overestimating the actual disease burden; (iii) animals whose diagnosis was based on clinical signs were also excluded from this study, which may have resulted in the omission of actual cases in resource‐limited settings where laboratory confirmation is not routinely available; (iv) this study included a limited number of published articles related to wild animals, which hampers inference on the role of wildlife in disease epidemiology; (v) heterogeneity in the models was significant, suggesting that other factors not considered might have had substantial effects; therefore, pooled estimates should be interpreted with caution; and (vi) the systematic review protocol was not prospectively registered in PROSPERO or any other public registry. However, the review was conducted according to a predefined methodology in accordance with PRISMA guidelines, including a systematic literature search, clearly defined inclusion and exclusion criteria, and a structured approach to data extraction and synthesis.

### 4.2. Policy Implications

The predominance of serotype O across Asia has important implications for vaccine development, underscoring the need for vaccines that provide robust protection against this serotype. However, regional variations in serotype prevalence indicate that immunization strategies should be tailored to local epidemiology. In countries such as Bangladesh, India, and Pakistan, the cocirculation of multiple serotypes (O, A, and Asia 1) underscores the need for polyvalent vaccines to achieve adequate control, particularly where continuous molecular characterization of circulating strains is limited. Understanding the sporadic occurrence of less common serotypes, such as SAT‐2 in Iraq, remains critical for anticipating outbreaks and implementing targeted interventions. Finally, these observations emphasize the importance of coordinated regional strategies, continuous surveillance, and adaptive vaccination policies to mitigate the risk of transboundary FMD transmission and optimize disease control in resource‐limited settings.

## 5. Conclusion

The systematic review and meta‐analysis conclude that FMD remains endemic across multiple Asian countries and continues to pose a significant transboundary threat, particularly affecting buffaloes. The disease exhibits considerable variability in prevalence across species, countries, diagnostic methods, and disease occurrence types, reflecting the complex epidemiology of FMD in the region. Serotype O was identified as the most widespread, followed by A, Asia‐1, and SAT‐2, highlighting the ongoing circulation of multiple FMDV serotypes. As a category A disease, FMD is considered highly dangerous because of its severe impact on the trade of live animals and animal products. To address this, both governmental bodies and livestock producers must implement consistent vaccination campaigns and effective management strategies to reduce overall disease prevalence and control contributing risk factors. There is also a clear need for further research into FMD, along with the dissemination of those findings. Combating the disease across Asia will require coordinated action among all stakeholders within the animal health sector across various nations. Additionally, strengthening disease surveillance, reporting, early detection, and rapid response systems is strongly recommended to manage and contain FMD outbreaks effectively.

## Author Contributions

Md Jisan Ahmed: conceptualization, investigation, data extraction and curation, formal analysis and interpretation of data, data visualization, writing–original draft, writing–review and editing, and project administration; Prajwal Bhandari and Md Imran Hossain: data extraction, data validation and curation, and writing–original draft; Ritu Chalise: writing–original draft; Md Ismile Hossain Bhuiyan and Md Arifur Rahman: data extraction, data curation, data validation, and writing–original draft; Kazi Estieque Alam: data visualization and data analysis; Israt Jahan Kaderi: data extraction and writing–original draft; Md Afiqul Islam Rahi and Tashmiah Tarin: data extraction and curation; Md Jahangir Alam: writing–review and editing; Ridwan Olamilekan Adesola: writing–review and editing; and Delower Hossain: data validation, curation and visualization, writing–original draft, and writing–review and editing.

## Funding

There was no funding for this study.

## Disclosure

All the authors read the full manuscript and agreed to its publication.

## Ethics Statement

The authors have nothing to report.

## Consent

The authors have nothing to report.

## Conflicts of Interest

The authors declare no conflicts of interest.

## Supporting Information

S1: PRISMA 2009 checklist.

S2: Quality assessment checklist.

S3: Frequencies of quality categories of the selected studies.

## Supporting information


**Supporting Information** Additional supporting information can be found online in the Supporting Information section.

## Data Availability

The datasets used and/or analyzed during the current study are available in the manuscript and supporting information.
