# Supplementary material for: Prevalence and Serotype Distribution of Foot and Mouth Disease (FMD) Virus in Asian Countries: A Systematic Review and Meta‐Analysis
Source: Vet Med Int. 2026 Feb 9;2026:5492291. doi: 10.1155/vmi/5492291 (PMC12884569; doi:10.1155/vmi/5492291)
Supplement: Supplementary file 1 — Supporting Information Additional supporting information can be found online in the Supporting Information section. [file VMI-2026-5492291-s001.docx]

**Prevalence and Serotype Distribution of Foot-and-Mouth Disease (FMD) Virus in Asian Countries: A Systematic Review and Meta-Analysis**

**Supplementary Materials**

**S1:** **PRISMA 2009 Checklist**

| **Section/topic** | **#** | **Checklist item** | **Reported on page #** |
| --- | --- | --- | --- |
| **TITLE** | | |  |
| Title | 1 | Identify the report as a systematic review, meta-analysis, or both. | 1 |
| **ABSTRACT** | | |  |
| Structured summary | 2 | Provide a structured summary including, as applicable: background; objectives; data sources; study eligibility criteria, participants, and interventions; study appraisal and synthesis methods; results; limitations; conclusions and implications of key findings; systematic review registration number. | 2 |
| **INTRODUCTION** | | |  |
| Rationale | 3 | Describe the rationale for the review in the context of what is already known. | 3-4 |
| Objectives | 4 | Provide an explicit statement of questions being addressed with reference to participants, interventions, comparisons, outcomes, and study design (PICOS). | 4 |
| **METHODS** | | |  |
| Protocol and registration | 5 | Indicate if a review protocol exists, if and where it can be accessed (e.g., Web address), and, if available, provide registration information, including registration number. | N/A |
| Eligibility criteria | 6 | Specify study characteristics (e.g., PICOS, length of follow-up) and report characteristics (e.g., years considered, language, publication status) used as criteria for eligibility, giving rationale. | 4-5 |
| Information sources | 7 | Describe all information sources (e.g., databases with dates of coverage, contact with study authors to identify additional studies) in the search and date last searched. | 4-5 |
| Search | 8 | Present a full electronic search strategy for at least one database, including any limits used, such that it could be repeated. | 4-5 |
| Study selection | 9 | State the process for selecting studies (i.e., screening, eligibility, included in the systematic review, and, if applicable, included in the meta-analysis). | 5 |
| Data collection process | 10 | Describe the method of data extraction from reports (e.g., piloted forms, independently, in duplicate) and any processes for obtaining and confirming data from investigators. | 6-9 |
| Data items | 11 | List and define all variables for which data were sought (e.g., PICOS, funding sources) and any assumptions and simplifications made. | 6-9 |
| Risk of bias in individual studies | 12 | Describe methods used for assessing risk of bias of individual studies (including specification of whether this was done at the study or outcome level), and how this information is to be used in any data synthesis. | N/A |
| Summary measures | 13 | State the principal summary measures (e.g., risk ratio, difference in means). | 10 |
| Synthesis of results | 14 | Describe the methods of handling data and combining results of studies, if done, including measures of consistency (e.g., I^2^) for each meta-analysis. | 10 |

Page 1 of 2

| **Section/topic** | **#** | **Checklist item** | **Reported on page #** |
| --- | --- | --- | --- |
| Risk of bias across studies | 15 | Specify any assessment of risk of bias that may affect the cumulative evidence (e.g., publication bias, selective reporting within studies). | 10 |
| Additional analyses | 16 | Describe methods of additional analyses (e.g., sensitivity or subgroup analyses, meta-regression), if done, indicating which were pre-specified. | 10 |
| **RESULTS** | | |  |
| Study selection | 17 | Give the number of studies screened, assessed for eligibility, and included in the review, with reasons for exclusions at each stage, ideally with a flow diagram. | 10-13 |
| Study characteristics | 18 | For each study, present characteristics for which data were extracted (e.g., study size, PICOS, follow-up period) and provide the citations. | 10-13 |
| Risk of bias within studies | 19 | Present data on the risk of bias of each study and, if available, any outcome-level assessment (see item 12). | N/A |
| Results of individual studies | 20 | For all outcomes considered (benefits or harms), present, for each study: (a) simple summary data for each intervention group (b) effect estimates and confidence intervals, ideally with a forest plot. | 15 |
| Synthesis of results | 21 | Present the results of each meta-analysis done, including confidence intervals and measures of consistency. | 15 |
| Risk of bias across studies | 22 | Present results of any assessment of risk of bias across studies (see Item 15). | 23-25 |
| Additional analysis | 23 | Give results of additional analyses, if done (e.g., sensitivity or subgroup analyses, meta-regression [see Item 16]). | 15-26 |
| **DISCUSSION** | | |  |
| Summary of evidence | 24 | Summarize the main findings, including the strength of evidence for each main outcome; consider their relevance to key groups (e.g., healthcare providers, users, and policy makers). | 28-31 |
| Limitations | 25 | Discuss limitations at study and outcome level (e.g., risk of bias), and at review-level (e.g., incomplete retrieval of identified research, reporting bias). | 31 |
| Conclusions | 26 | Provide a general interpretation of the results in the context of other evidence, and implications for future research. | 31 |
| **FUNDING** | | |  |
| Funding | 27 | Describe sources of funding for the systematic review and other support (e.g., supply of data); role of funders for the systematic review. | 32-33 |

*From:*  Moher D, Liberati A, Tetzlaff J, Altman DG, The PRISMA Group (2009). Preferred Reporting Items for Systematic Reviews and Meta-Analyses: The PRISMA Statement. PLoS Med 6(7): e1000097. doi:10.1371/journal.pmed1000097

For more information, visit: [**www.prisma-statement.org**](http://www.prisma-statement.org).

Page 2 of 2

**Text S2:** Quality assessment checklist

The following items were examined and given a score based on a simple scale system (1 for ''yes'', 0 for ''no'').

1. Was the research objective clearly stated?
2. Was the sampling area clearly described with reference to the location?
3. Was the period of the study stated?
4. Was some form of random selection used to select the samples?
5. Was a minimum sample size calculated?
6. Were the sample processing and diagnostic methods clearly described?
7. Was the target population (Species of animals) clearly mentioned?

The quality index score for each study was calculated by dividing the study quality score by 7.

**Table S3: Quality score and quality index score of individual contributing studies**

| **Serial** | **Author** | **Year** | **Quality Score** | **Quality Index core** |
| --- | --- | --- | --- | --- |
| 1 | (Giasuddin et al., 2016) | 2016 | 7 | 1.00 |
| 2 | (Nandi et al., 2015) | 2015 | 7 | 1.00 |
| 3 | (Siddique et al., 2018) | 2018 | 7 | 1.00 |
| 4 | (Rout et al., 2014) | 2013 | 7 | 1.00 |
| 5 | (Farooq et al., 2017) | 2017 | 7 | 1.00 |
| 6 | (Mohanty et al., 2015) | 2015 | 6 | 0.86 |
| 7 | (Nawaz et al., 2018) | 2018 | 6 | 0.86 |
| 8 | (Hegde et al., 2016) | 2016 | 7 | 1.00 |
| 9 | (Ullah et al., 2020). | 2020 | 7 | 1.00 |
| 10 | (Buckle et al., 2021) | 2021 | 6 | 0.86 |
| 11 | (Singh et al., 2020) | 2020 | 6 | 0.86 |
| 12 | (Dhakal et al., 2023). | 2023 | 7 | 1.00 |
| 13 | (Dhurba et al., 2024) | 2024 | 6 | 0.86 |
| 14 | (Dukpa et al., 2011) | 2011 | 7 | 1.00 |
| 15 | (Mahmoud & Galbat, 2017) | 2017 | 4 | 0.57 |
| 16 | (Mahmoud et al., 2017) | 2017 | 7 | 1.00 |
| 17 | (Hussain et al., 2019) | 2019 | 5 | 0.71 |
| 18 | (Lignereux et al., 2020) | 2020 | 4 | 0.57 |
| 19 | (Enad & Mansour, 2025) | 2025 | 4 | 0.57 |
| 20 | (Abd Hatem et al., 2022) | 2022 | 6 | 0.86 |
| 21 | (Al-Rodhan, 2014) | 2014 | 6 | 0.86 |
| 22 | (Gadir et al., 2023) | 2023 | 6 | 0.86 |
| 23 | (Tum et al., 2015) | 2015 | 6 | 0.86 |
| 24 | (Kong et al., 2023) | 2023 | 7 | 1.00 |
| 25 | (Lim et al., 2022) | 2022 | 3 | 0.43 |
| 26 | (Mahmoud et al., 2021) | 2021 | 7 | 1.00 |
| 27 | (Azimi et al., 2020) | 2020 | 3 | 0.43 |
| 28 | (Park et al., 2016) | 2015 | 3 | 0.43 |
| 29 | (Khan et al., 2024) | 2024 | 6 | 0.86 |
| 30 | (Singanallur et al., 2020) | 2020 | 7 | 1.00 |
| 31 | (Siengsanan-Lamont et al., 2021) | 2021 | 6 | 0.86 |
| 32 | (Xaydalasouk et al., 2021) | 2021 | 5 | 0.71 |
| 33 | (Gee et al., 2024) | 2024 | 7 | 1.00 |
| 34 | (Siengsanan-Lamont et al., 2022) | 2022 | 6 | 0.86 |
| 35 | (Holt et al., 2019) | 2019 | 5 | 0.71 |
| 36 | (Sulistyaningrum et al., 2024) | 2024 | 5 | 0.71 |
| 37 | (Jauhari et al., 2024) | 2024 | 3 | 0.43 |
| 38 | (Abubakar & Manzoor, 2013) | 2013 | 3 | 0.43 |
| 39 | (Khan et al., 2016) | 2016 | 7 | 1.00 |
| 40 | (MacPhillamy et al., 2022) | 2021 | 7 | 1.00 |
| 41 | (Ranabijuli et al., 2010) | 2010 | 6 | 0.86 |
| 42 | (Osmani et al., 2019) | 2019 | 4 | 0.57 |
| 43 | (Osmani et al., 2021) | 2021 | 7 | 1.00 |
| 44 | (Albayrak et al., 2017) | 2017 | 4 | 0.57 |
| 45 | (Ince, 2019) | 2019 | 3 | 0.43 |
| 46 | (Hossain et al., 2023) | 2023 | 4 | 0.57 |
| 47 | (Alam et al., 2015) | 2015 | 4 | 0.57 |
| 48 | (Loth et al., 2011) | 2010 | 4 | 0.57 |
| 49 | (Ranjan et al., 2018) | 2018 | 4 | 0.57 |
| 50 | (Hayer et al., 2018) | 2018 | 6 | 0.86 |
| 51 | (Borah et al., 2018) | 2018 | 5 | 0.71 |
| 52 | (Rout et al., 2016) | 2016 | 6 | 0.86 |
| 53 | (Klein et al., 2008) | 2008 | 6 | 0.86 |
| 54 | (Ahmed et al., 2018) | 2018 | 7 | 1.00 |
| 55 | (Farooq et al., 2018) | 2018 | 6 | 0.86 |
| 56 | (Saeed et al., 2011) | 2011 | 7 | 1.00 |
| 57 | (Waheed et al., 2011) | 2011 | 6 | 0.86 |
| 58 | (Kabir et al., 2024) | 2024 | 6 | 0.86 |
| 59 | (Jamal et al., 2012) | 2011 | 6 | 0.86 |
| 60 | (Zhang et al., 2023) | 2023 | 6 | 0.86 |
| 61 | (Wang et al., 2018) | 2018 | 3 | 0.43 |
| 62 | (de Carvalho Ferreira et al., 2017) | 2015 | 7 | 1.00 |
| 63 | (Gunasekara et al., 2021) | 2021 | 7 | 1.00 |
| 64 | (Qiu et al., 2018) | 2018 | 6 | 0.86 |
| 65 | (Najafi et al., 2020) | 2020 | 5 | 0.71 |
| 66 | (Rashtibaf et al., 2012) | 2012 | 7 | 1.00 |
| 67 | (Lim et al., 2020) | 2020 | 4 | 0.57 |
| 68 | (Mansour et al., 2018) | 2018 | 5 | 0.71 |

**Figure 1 S1: Frequency of quality category of selected studies**

**Table S4.** Search Strings used for this systematic review and meta-analysis

| **Data bases** | **Search strings** | **Filters** |
| --- | --- | --- |
| PubMed | (“Prevalence” OR “Seroprevalence” OR “Incidence” OR “Frequency” OR “Occurrence” OR “Characterization” OR “Epidemiology”) AND (“FMD” OR “Foot-and-Mouth Disease”) | English language; publication dates January 2008 – February 2025; article types |
| Google Scholar | (“Prevalence” OR “Seroprevalence” OR “Incidence” OR “Frequency” OR “Occurrence” OR “Characterization” OR “Epidemiology”) AND (“FMD” OR “Foot-and-Mouth Disease”) |  |
| Springer Link | (“Prevalence” OR “Seroprevalence” OR “Incidence” OR “Frequency” OR “Occurrence” OR “Characterization” OR “Epidemiology”) AND (“FMD” OR “Foot-and-Mouth Disease”) |  |
| ScienceDirect | (“Prevalence” OR “Seroprevalence” OR “Incidence” OR “Frequency” OR “Occurrence” OR “Characterization” OR “Epidemiology”) AND (“FMD” OR “Foot-and-Mouth Disease”) |  |
